# Enhanced YOLO and Scanning Portal System for Vehicle Component Detection

**DOI:** 10.3390/s25154809

**Published:** 2025-08-05

**Authors:** Feng Ye, Mingzhe Yuan, Chen Luo, Shuo Li, Duotao Pan, Wenhong Wang, Feidao Cao, Diwen Chen

**Affiliations:** 1 College of Information Engineering, Shenyang University of Chemical Technology, Shenyang 110142, China; yefeng@gz.sia.cn (F.Y.); luocheng@gz.sia.cn (C.L.); panduotao@syuct.edu.cn (D.P.); 2 Guangzhou Institute of Industrial Intelligence, Guangzhou 511458, China; lishuo@gz.sia.cn (S.L.); wangwenhong@gz.sia.cn (W.W.); caofeidao@sia.cn (F.C.); chendiwen@gz.sia.cn (D.C.); 3 Shenyang Institute of Automation, Chinese Academy of Sciences, Shenyang 110169, China

**Keywords:** target detection, automobile parts inspection, system design, YOLOv12n

## Abstract

In this paper, a novel online detection system is designed to enhance accuracy and operational efficiency in the outbound logistics of automotive components after production. The system consists of a scanning portal system and an improved YOLOv12-based detection algorithm which captures images of automotive parts passing through the scanning portal in real time. By integrating deep learning, the system enables real-time monitoring and identification, thereby preventing misdetections and missed detections of automotive parts, in this way promoting intelligent automotive part recognition and detection. Our system introduces the A2C2f-SA module, which achieves an efficient feature attention mechanism while maintaining a lightweight design. Additionally, Dynamic Space-to-Depth (Dynamic S2D) is employed to improve convolution and replace the stride convolution and pooling layers in the baseline network, helping to mitigate the loss of fine-grained information and enhancing the network’s feature extraction capability. To improve real-time performance, a GFL-MBConv lightweight detection head is proposed. Furthermore, adaptive frequency-aware feature fusion (Adpfreqfusion) is hybridized at the end of the neck network to effectively enhance high-frequency information lost during downsampling, thereby improving the model’s detection accuracy for target objects in complex backgrounds. On-site tests demonstrate that the system achieves a comprehensive accuracy of 97.3% and an average vehicle detection time of 7.59 s, exhibiting not only high precision but also high detection efficiency. These results can make the proposed system highly valuable for applications in the automotive industry.

## 1. Introduction

### 1.1. Motivation

With the continuous advancement of intelligence and automation in the automotive industry, precise inspection technology for automotive components has become one of the core requirements in manufacturing, maintenance, and quality control. In the context of smart manufacturing, ensuring dimensional accuracy and assembly consistency of components directly impacts the reliability and safety of vehicles. In full-cycle quality management, end-to-end inspection data from raw materials to finished products provides a scientific basis for process optimization and product iteration. Therefore, the innovation and application level of automotive component inspection technology has become a key indicator for measuring industrial competitiveness.

Automotive component inspection primarily falls into two categories: manual inspection and machine vision inspection. Traditional manual inspection relies on experienced technicians, yet is susceptible to subjective influences and inefficiency. Statistical data indicate that skilled workers in mainstream automotive manufacturers spend an average of 10–15 min on quality inspection per vehicle, with the missed detection rate for body panel components increasing significantly when working for extended periods. Consequently, this approach struggles to meet the demands of the modern automotive industry [1].

Machine vision inspection mainly consists of traditional methods and deep learning-based methods. In the detection and recognition of automotive components, traditional methods rely on conventional image processing techniques, including image segmentation [2], template matching [3], feature point matching [4,5,6], and binocular vision positioning [7]. These methods perform acceptably in structured environments or simple scenarios. However, in complex industrial settings, their generalization capability is significantly constrained due to diverse component geometries, limited lighting conditions, background interference, and other factors. Moreover, the feature extraction ability of traditional algorithms can no longer meet the demands of highly dynamic environments. To address these challenges, in this study we design a deep learning-based visual inspection system with optimized algorithms to fulfill the practical requirements of industrial applications.

### 1.2. Related Work

The rapid development of deep learning has led deep learning-based detection methods achieving significantly improved feature extraction capabilities and accuracy in industrial assembly applications. Krizhevsky et al. pioneered the application of deep learning in wear particle identification by proposing the AlexNet convolutional neural network for automotive bearing wear recognition in 2017 [8]. To reduce dependency on large training datasets, Peng et al. first introduced transfer learning into a CNN-based automotive wear particle identification system [9]. Wang Fei et al. addressed the limited discriminative ability of single neural networks for similar wear particles by combining BP neural networks with CNN algorithms, achieving over 15% improvement in recognition accuracy [10].

In 2023, Jan Flusser’s team employed EfficientNet for wheel hub classification and dimensional inspection, leveraging ImageNet-based enhancements to achieve a final classification accuracy of 98.72% [11,12]. Muriel Mazzetto’s team tackled the challenges of manual brake kit inspection errors and the inability of traditional vision systems to adapt to rapid multi-model part switching in automotive assembly lines. By integrating SSD with MobileNet through the TensorFlow API and implementing a multi-frame voting mechanism to handle occlusion, they achieved 99.9% detection accuracy for six brake components, with a maximum recall of 91%. In the same year, to address traditional vision systems’ difficulty in segmenting automotive parts, they adopted DeepLabV3+ with atrous convolution and encoder–decoder structures to capture multi-scale features. This approach reached a mean IoU of 78.94% for cylinder head machining surfaces and holes, although performance remained limited for targets below 5 mm^2^ [13].

To address poor model adaptability when applying CLIP directly to industrial inspection, in 2020 Fadel M. Megahed’s team utilized CLIP’s visual encoder for few-shot learning. By comparing test images with limited training samples via cosine similarity of embedded vectors, their method achieved high accuracy in five industrial scenarios (e.g., metal disk inspection, STS detection, microstructure classification), including STS detection targets with stochastic textured surfaces such as woven textiles and metrology data of machined metal surface, which are challenging for traditional methods due to their inherent randomness. The CLIP-based few-shot approach showed strong performance, especially with the ViT-L/14 encoder. It achieved 0.97 accuracy with 50 examples per class, although performance degraded in complex multi-component scenarios [14,15]. In 2023, Mohammed Salah’s team resolved issues with neuromorphic vision sensors’ sensitivity to motion blur and lighting in automated assembly lines by combining neuromorphic techniques, event compensation/clustering algorithms, and robust circle detection with the Huber loss and Levenberg–Marquardt optimization. This approach improved detection efficiency tenfold compared to traditional methods while meeting industrial requirements [16].

This study contributes the following improvements:

1. A scanning portal system for automotive part detection that assists workers in efficient inspection using character labels, colors, and shapes, thereby reducing workload.
2. Due to comparative analysis showing that YOLOv12n excels in complex motion scenarios and dynamic multi-object detection, to meet the needs of real-time inspection of automotive components, we use YOLOv12 as a baseline and introduce the A2C2f-SA module for efficient attention to features. We further optimize the convolutional structure with Dynamic S2D, replacing stride convolutions and pooling layers to reduce fine-grained information loss. Additionally, the lightweight GFL-MBConv detection head improves real-time performance, while integration of the Adpfreqfusion module at the network’s neck enhances high-frequency information retention. These innovations significantly improve detection accuracy and efficiency in real-world In Proceedings of theindustrial applications.
3. To fill gaps in current industrial datasets, a dataset of 10,070 automotive component images is provided to address misdetections, missed detections, and inefficiencies in manual inspection. This dataset is available from the authors upon request.

The remainder of this paper is structured as follows:

- Section 2 details the scanning portal system architecture, emphasizing YOLO network improvements and dataset construction.
- Section 3 presents experimental evaluations of the enhanced model and system.
- Section 4 analyzes the results and validates detection effectiveness.
- Section 5 concludes with research summaries and future directions.

## 2. Materials and Methods

### 2.1. Scanning Portal System Device

#### 2.1.1. Scanning Portal System Introduction

To meet the requirements for precise outbound logistics of automotive components after production, the production line employs an intelligent recognition and inspection system based on machine vision technology. This system performs automated detection of components placed on Automated Guided Vehicle (AGV) transport racks during transfer to the shipping handover area.

The system utilizes an intelligent visual recognition module to monitor components on the racks without disrupting normal logistics workflows. This allows logistics personnel to focus on transportation tasks while delegating more accurate and efficient identification operations to the intelligent system. The system captures multi-angle images via industrial cameras, enabling intelligent recognition of various automotive parts through barcode labels and component features, ensuring that the placement of parts on the racks strictly complies with production instructions. All identification data is uploaded in real-time to the enterprise server and synchronized with the Warehouse Management System (WMS).

When placement anomalies are detected, the system immediately alerts relevant personnel and automatically generates comprehensive exception records. While maintaining production safety, the system fulfills all inspection requirements within the production cycle through data acquisition, AI model-based real-time detection, and other advanced technologies. Finally, all inspection data are systematically organized, analyzed, and stored to establish a complete traceability system, preventing misplacement or omission errors when racks are delivered to the same production line.

During operation, the intelligent recognition module continuously monitors the models of automotive components on AGV transport racks and cross-references them in real-time with outbound order data from the WMS. All scanning results are uploaded to the central management system in standardized data formats. In case of system anomalies such as crashes or recognition module failures, operators can use backup barcode scanners for manual verification, ensuring uninterrupted production flow.

**Figure 1:**
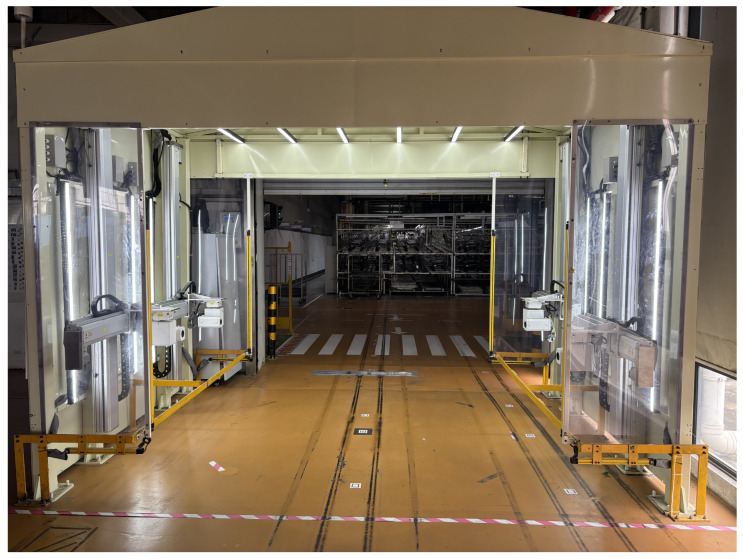
Scanning portal system.

The implementation of this system has significantly improved outbound accuracy, eliminated incorrect shipments, and provided reliable quality assurance for automotive component manufacturing. The scanning portal system is shown in Figure 1.

#### 2.1.2. Scanning Portal System Composition Framework

**Figure 2:**
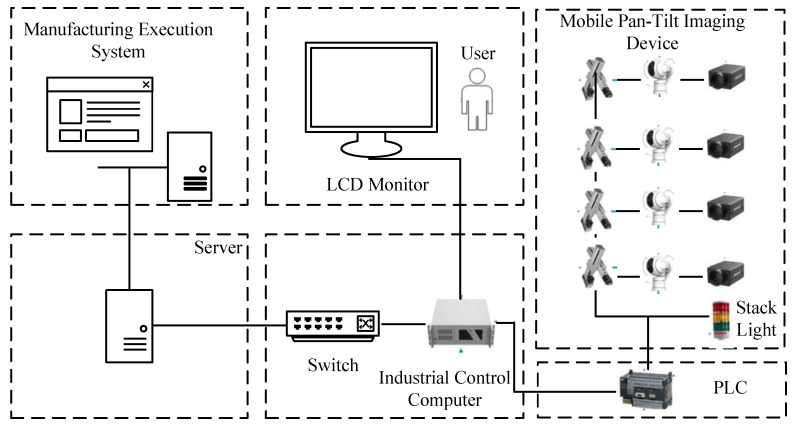
Scanning portal system composition framework.

The overall framework of the scanning portal system is shown in Figure 2.

**Figure 3:**
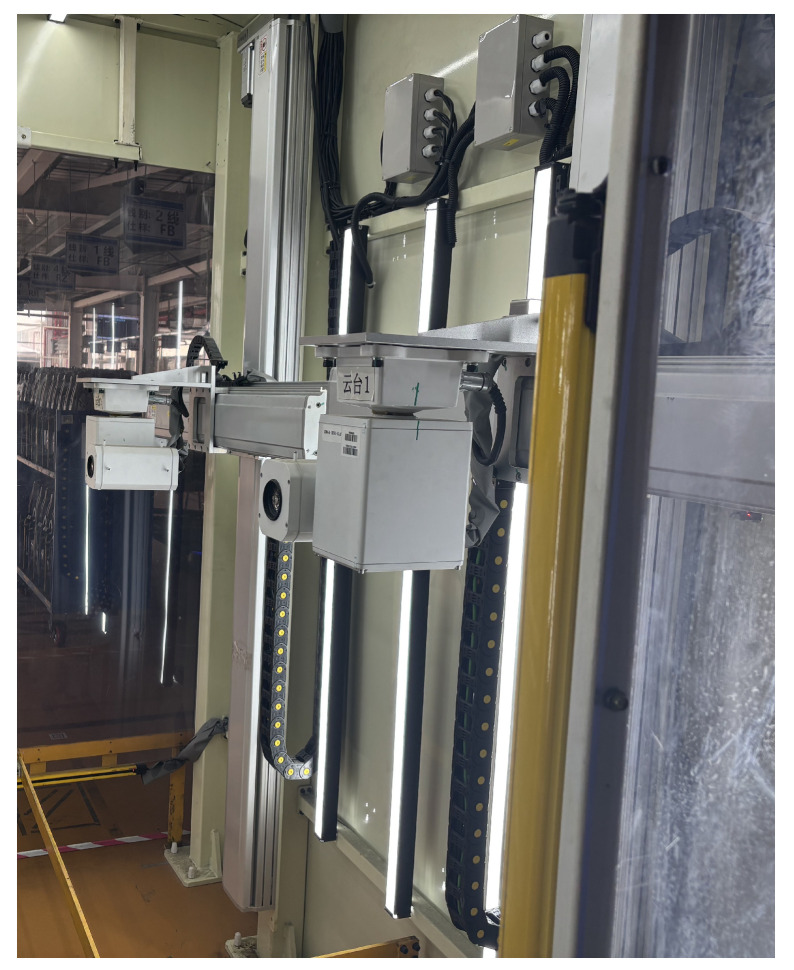
Mobile pan–tilt imaging device.

**Figure 4:**
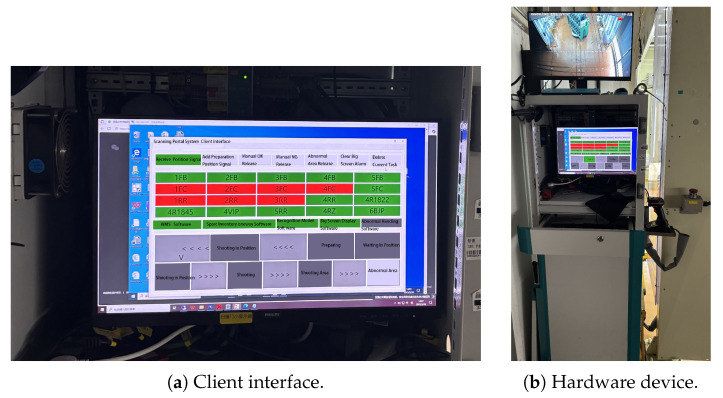
System components overview: (**a**) client interface showing the operational dashboard and (**b**) hardware setup of the scanning portal system. All components are designed for industrial-grade reliability.

The main components of the scanning portal system include the following:

- Photography Studio: Designed to provide stable and sufficient lighting conditions for the shooting process. Dark-colored photographic cloth is used to cover the studio and column areas, isolating external light interference. High-power LED light sources are employed around the studio to create a stable internal lighting environment.
- Mobile Pan–Tilt Imaging Device: To effectively collect images of automotive parts placed on AGV transport racks, four cameras integrated with motion modules are installed on both sides of the photography studio. The cameras can move up and down and rotate left and right through the motion modules to adjust shooting angles, thereby obtaining clear image data. The mobile pan–tilt imaging device is shown in Figure 3, while the physical system hardware is shown in Figure 4.
- Client: Integrated with an industrial control computer, monitor, and alarm module, it implements a comprehensive human–machine interaction interface developed based on the OT6.5 framework. The client has the following three core functions:
  1. Automatic detection and recognition of assembly components through image processing algorithms.
  2. Real-time visualization of detection results.
  3. Immediate alarm response to abnormal situations.
- WMS Communication System and Hardware: This system is responsible for receiving, processing, and storing data from imaging devices and feedback information from the client. It controls the pan–tilt movement and shooting of the camera by sending signals to activate the Programmable Logic Controller (PLC).

### 2.2. Model Selection

#### 2.2.1. YOLOv12 Network

The YOLO series of detection algorithms has been widely applied in the field of object detection due to its high accuracy and detection efficiency. YOLOv12 was released on 19 February 2025. Unlike previous YOLO algorithms that focused on improvements to CNNs, YOLOv12 primarily revolves around enhancements to the regional attention mechanism. Its network architecture mainly consists of four parts: the input layer, backbone, neck, and head. After preprocessing at the input layer, the image undergoes feature extraction through the backbone followed by fusion and enhancement via the neck network, before being sent to the detection head for output [17].

YOLOv12n innovatively adopts the A2C2f module based on the regional attention mechanism, which divides the feature map into equal-sized regions either horizontally or vertically. This effectively handles large receptive fields while significantly reducing computational costs compared to standard self-attention. Additionally, it redesigns the standard attention mechanism by employing flash attention to minimize memory access overhead, adjusting the MLP ratio to balance computation between the attention layer and the feedforward layer, and incorporating a 7 × 7 separable convolution into the attention mechanism for implicit positional encoding. Furthermore, YOLOv12n utilizes the Residual Efficient Layer Aggregation Network (R-ELAN) for feature aggregation. This introduces block-level residual connections with scaling capabilities and redesigns the feature aggregation method to create a bottleneck-like structure.

#### 2.2.2. Enhanced YOLOv12 Network

In the field of automotive component recognition and detection, existing models currently face challenges such as difficult feature extraction due to limited lighting sampling conditions and construction vibrations, boundary blur, and lack of precise high-frequency details caused by simple upsampling as well as high requirements for on-site computing resources. Even the mainstream YOLO models exhibit less than ideal detection performance. Therefore, this paper takes YOLOv12 as the baseline network and proposes improvements.

First, the Conv module is enhanced by integrating Dynamic Space-to-Depth (Dynamic S2D) to replace the original convolution module and pooling layer, enabling the network to better extract features from low-resolution inputs.

Second, the neck network is integrated with Adpfreqfusion (adaptive frequency domain-aware feature fusion), which enhances the model’s ability to extract features in complex backgrounds.

Third, to improve the network’s response speed and target focusing ability, the A2C2f-SA module is designed and incorporated to enhance information interaction and improve feature extraction efficiency.

**Figure 5:**
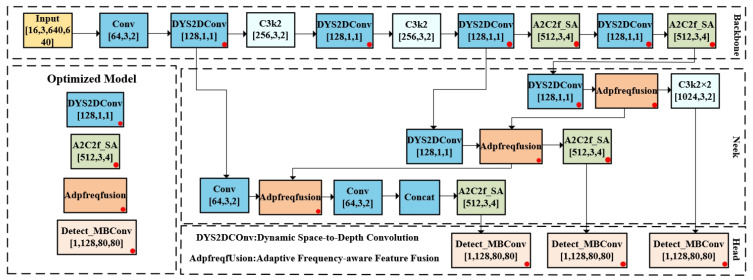
Enhanced YOLOv12n network structure.

Finally, to address the real-time deployment issue, the traditional multi-parameter detection head is replaced with the GFL-MBConv lightweight detection head, which reduces computational costs while maintaining good detection performance. The improved YOLOv12n network structure is shown in Figure 5.

#### 2.2.3. Dynamic Space-to-Depth Convolution Module

**Figure 6:**
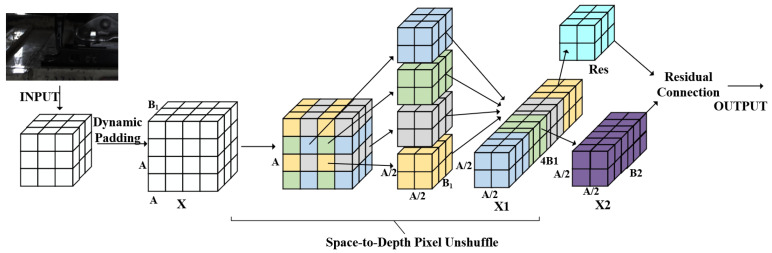
Extraction process of DYS2DConv.

In practical imaging scenarios, the AGV-transported racks are divided into upper, middle, and lower tiers, with varying numbers of components on each shelf layer, resulting in complex backgrounds that hinder effective target feature extraction. To address this issue, Dynamic Spatial-to-Depth (Dynamic S2D) rearrangement is integrated to optimize the original convolutional layer, thereby enhancing the network’s feature extraction capability. The resulting Dys2dConv primarily consists of a Dynamic S2D layer and a non-strided convolutional residual layer:

- Dynamic S2D layer (DYS2D): This layer consists of an operation that realizes spatial information recombination and downsampling by combining the checkerboard splitting method with channel rearrangement. During the downsampling process, this layer retains all information to the greatest possible extent. For any feature map *X* (with a size of $A\times A\times B_{1}$), this layer divides it into ${\mathrm{Scale}}^{2}$ sub-maps (each with a size of $A/\mathrm{Scale}\times A/\mathrm{Scale}\times B_{1}$) according to the scale (downsampling multiple). If the size of the feature map is not an integer multiple of the downsampling multiple, then a symmetric padding strategy is adopted to fill the feature map, which overcomes the boundary information redundancy caused by the inability of the original SPD layer to process feature maps with non-divisible downsampling multiples. Each sub-map also takes specific position pixel values, with their corresponding probability distributions belonging to different blocks according to the Scale. Softmax is used for normalization. All sub-maps are then concatenated along the channel dimension and corresponding probabilities to form a new feature map $X_{1}$, achieving adaptive rearrangement. Compared with *X*, the spatial dimension of $X_{1}$ is reduced by a factor of Scale, while the channel dimension is correspondingly increased by a factor of ${\mathrm{Scale}}^{2}$. Through such a transformation, lossless conversion of spatial information to channel information is realized. The correspondence between the elements in the new feature map $X_{1}$ and those in the original feature map *X* is shown in Equation (1), where *i* and *j* are the spatial coordinates of the output feature map, *k* is the channel index of the output feature map, and *C* is the number of channels. The specific form of the softmax probability distribution is shown in Equation (2), where $P_{i,j,k}$ represents the softmax probability at position (i,j) for channel *k* and the summation in the denominator is computed over all spatial positions (m,n) in the input feature map for the same channel *k*:
  (1) $$\begin{array}{c} {X1}_{\left\{i,j,k\right\}}=X_{\left\{i\cdot scale\;+\;\left(k\;÷C\right),\;j\cdot scale\;+\;\left(k\;mod\;C\right)\right\}}, \end{array}$$
  (2) $$\begin{array}{c} P_{i,j,k}=\frac{\exp(X_{i,j,k})}{\sum_{m,n}\exp\left(X_{m,n,k}\right)}. \end{array}$$
- Non-Strided Convolutional Residual Layer: After the feature map *X* is transformed into $X_{1}$ through the SPD layer, a non-strided convolutional layer (stride = 1) with $B_{2}$ filters (satisfying $B_{2}<B_{1}\times {\mathrm{Scale}}^{2}$) is introduced to convert $X_{1}$ into $X_{2}$. Although using filters with a stride greater than 1 can superficially complete the transformation from $X_{1}$ to $X_{2}$, the problem of non-discriminative loss is prone to occur due to the different sampling frequencies of even and odd rows/columns in the feature map. Additionally, using a non-strided convolutional layer avoids the feature shrinkage issue caused by filters with a stride greater than 1. When the number of input channels $c_{1}$ is inconsistent with the number of output channels $c_{2}$, a residual branch is employed to adjust the channels of $X_{1}$ to obtain the residual term res. Finally, $X_{2}$ is added to the residual term and activated by the SILU function for output. The extraction process of DYS2DConv is shown in Figure 6. The core pseudocode of DYS2DConv is implemented as follows:
  $$\begin{array}{c} Out=(attn\_map\_down.unsqueeze(2)*blocks).sum(dim=1). \end{array}$$
  Unlike standard S2D, weighted summation is performed on sub-pixel blocks rather than simple concatenation or rearrangement, resulting in enhanced information selectivity.

#### 2.2.4. A2C2f-SA Module

To improve the network’s response speed and target focusing ability, the regional attention mechanism in the original A2C2f module is replaced with the more concise ShuffleAttention [18]. This attention mechanism effectively aggregates two widely used attention mechanisms, namely, channel attention and spatial attention. The former aims to capture features of key channels, while the latter focuses on enhancing the features of critical positions. The core operation of A2C2f-SA is expressed via the following pseudocode:

$$\begin{array}{c} y\_new=ShuffleAttention(y\_list[-1]),\\ y\_new=ShuffleAttention(y\_new). \end{array}$$

**Figure 7:**
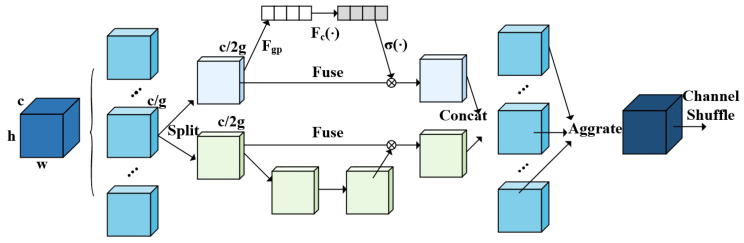
Extraction process of ShuffleAttention.

Employing two consecutive ShuffleAttention operations in place of a single C3k module promotes information interaction between different groups, helping to avoid information isolation. ShuffleAttention mainly consists of the following three steps:

1. Feature Grouping: Any feature map $X\in R^{C\times H\times W}$ (where C, H, and W denote the number of channels, spatial height, and width, respectively) is divided into G groups; the number of channels in each group is $C_{1}=C/G$, and the G sub-feature maps after grouping are expressed as Equation (3):
  (3) $$X=\left[X_{1},X_{2},X_{3},\ldots ,X_{G}\right]\;X_{i}\in R^{C1\times H\times W}.$$
2. Mixed Attention: Subsequently, channel attention and spatial attention are calculated for each sub-feature map $X_{i}$ and the results are fused. The channel attention is computed as shown in Equation (4), while the spatial attention is computed as shown in Equation (5):
  (4) $$\mathrm{CA}\left(X_{i}\right)=\sigma \left(W_{1}\cdot \mathrm{GAP}\left(X_{i}\right)\right)⊙X_{i},$$
  (5) $$SA\left(X_{i}\right)=\sigma \left(Conv\left(X_{i}\right)\right)⊙X_{i}.$$
3. Channel Shuffling: Finally, the outputs of different groups $Y=[Y_{1},Y_{2},\ldots ,Y_{G}]$ are rearranged according to $Y^{\prime }=\mathrm{Shuffle}\left[Y\right]$ to obtain $Y^{\prime }$, enabling information interaction between different groups, where Shuffle represents the channel shuffling operation. The specific workflow of the A2C2f-SA attention mechanism is illustrated in Figure 7, where $F_{\mathrm{gp}}$ is defined by Equation (6) and $F_{c}\left(x\right)$ is defined by Equation (7). Here, $W^{\prime }$ controls the contribution weights of different input features to the output and b denotes the base offset for each output dimension:
  (6) $$F_{gp}\left(X_{k1}\right)=\frac{1}{H\times W}\sum_{i=1}^{H}\sum_{j=1}^{W}X_{k1}\left(i,j\right),$$
  (7) $$F_{c}\left(x\right)=W^{\prime }x+b.$$

#### 2.2.5. Adaptive Frequency-Aware Feature Fusion for Better Fusion Solutions

Frequency-aware feature fusion was first proposed by Linwei Chen et al. in 2024 [19]. Its purpose is to address the fusion problems that occur when fusing low-resolution and high-frequency features with high-resolution and low-frequency features. In this problem, the fusion feature values change due to the inconsistency of features for the same type of targets [19]. However, because fusion of high- and low-frequency features basically relies on convolutional kernels and static filtering, leaving it unable to learn the importance of high- and low-frequency information, we introduce an AdaptiveFreqGate. This enables each input to learn the gating to dynamically control the proportion of high- and low-frequency information, thereby enhancing the discriminative power of the fused features. Before fusing high- and low-frequency features, the features are first subjected to the Discrete Cosine Transform (DCT), as shown in Equation (8), then the weights of each frequency band are judged successively according to Equation (9):

(8) $$\begin{array}{cc} \tilde{z_{in}}\left(d,k_{1}\right) & =\left\{\begin{array}{cc} z_{in}\left(d,k_{1}\right), & 0\leq k_{1}\leq K\\ z_{in}\left(d,2K-k_{1}-1\right), & K<k_{1}\leq 2K-1, \end{array}\right. \end{array}$$

(9) $$\begin{array}{cc} \Delta W_{foura} & =F^{-1}\left(B\cdot G\left(z_{tr}\right)\cdot F\left(A\right)\right). \end{array}$$

In Equation (8), $\tilde{z_{in}}\left(d,k_{1}\right)$ represents the signal after symmetric extension transformation, $z_{in}$ represents the original input signal, *K* represents the effective length of the original signal, *d* represents the channel dimension, and $k_{1}$ represents the spatial—domain index of the signal. In Equation (9), $\Delta W_{foura}$ is the weight of the FouRA adapter, $F^{-1}$ represents the inverse Fourier transform, *B* is the frequency domain-filtering matrix, $G(z_{lr})$ represents the gating function, and *A* is the original feature basis.

The core pseudocode of Adpfreqfusion is implemented as follows:

$$\begin{array}{c} compressed\_hr=AdaptiveFreqGate(compressed\_hr),\\ compressed\_lr=AdaptiveFreqGate(compressed\_lr). \end{array}$$

**Figure 8:**
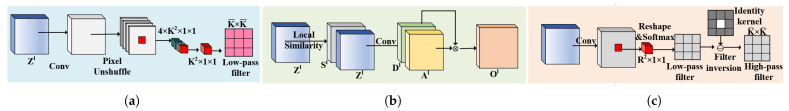
The three basic components constituting the AdpFreqFusion module: (**a**) adaptive low-pass filter, (**b**) offset generator, and (**c**) adaptive high-pass filter.

The core concept of the pseudocode employs gating mechanisms to enhance selective emphasis on critical frequency components while suppressing non-essential elements. As shown in Figure 8, the Adpfreqfusion module consists of three basic components: an Adaptive Low-Pass Filter (ALPF) used to smooth high-frequency features to mitigate the situation of constantly changing fusion feature values, as shown in Figure 8a; an Offset Generator used to correct large-area inconsistent features and optimize thin regions and boundary regions, as shown in Figure 8b; and an Adaptive High-Pass Filter (AHPF) used to enhance boundary information and retain high-resolution and accurate features, as shown in Figure 8c.

Adpfreqfusion feature fusion is mainly achieved through two stages, namely, adaptive initial fusion and final fusion. In the general initial fusion process, high-frequency and low-frequency information is fused and compressed as shown in Equation (10) to obtain $Z^{l}$ in order to provide efficient features to the three generators. However, high-resolution information merged in this way will have problems such as blurred boundaries and class inconsistency, meaning that it is still necessary to combine the adaptive low-pass filter, offset generator, and high-pass filter for enhanced fusion. The fusion operation is shown in Equations (11)–(18).

(10) $$\begin{array}{cc} Z^{l} & =F_{UP}\left({\mathrm{Conv}}_{1\times 1}\left(Y^{l+1}\right)\right)+{\mathrm{Conv}}_{1\times 1}\left(X^{l}\right) \end{array}$$

(11) $$\begin{array}{cc} {\bar{V}}^{l} & ={\mathrm{Conv}}_{3\times 3}\left(Z^{l}\right) \end{array}$$

(12) $$\begin{array}{cc} {\bar{W}}_{i,j}^{l,p,q} & =\mathrm{Softmax}\left(\frac{\exp({\bar{V}}_{i,j}^{l,p,q})}{\sum_{p,q\in \Omega }\exp\left({\bar{V}}_{i,j}^{l,p,q}\right)}\right) \end{array}$$

(13) $$\begin{array}{cc} {\tilde{Y}}_{i,j}^{l+1,g} & =\sum_{p,q\in \Omega }{\bar{W}}_{i,j}^{l,g,p,q}\cdot Y_{i+p,j+q}^{l+1} \end{array}$$

(14) $$\begin{array}{cc} {\tilde{Y}}^{l+1} & =\mathrm{PixelShuffle}\left({\tilde{Y}}_{i+1}^{l+1},{\tilde{Y}}_{i+2}^{l+1},{\tilde{Y}}_{i+3}^{l+1},{\tilde{Y}}_{i+4}^{l+1}\right) \end{array}$$

(15) $$\begin{array}{cc} S_{i,j}^{l,p,q} & =\frac{\sum_{c=1}^{C}Z_{c,i,j}^{l}\cdot Z_{c,i+p,j+q}^{l}}{\sqrt{\sum_{c=1}^{C}{\left(Z_{c,i,j}^{l}\right)}^{2}}\sqrt{\sum_{c=1}^{C}{\left(Z_{c,i+p,j+q}^{l}\right)}^{2}}} \end{array}$$

(16) $$\begin{array}{cc} X_{F}\left(u,v\right) & =\frac{1}{H\times W}\sum_{h=0}^{H-1}\sum_{w=0}^{W-1}X\left(h,w\right)e^{-2\pi j\frac{uh+vw}{HW}} \end{array}$$

(17) $$\begin{array}{cc} {\hat{W}}_{i,j}^{l,p,q} & =E-\mathrm{Softmax}\left({\bar{V}}_{i,j}^{l}\right)=E-\frac{\exp({\bar{V}}_{i,j}^{l,p,q})}{\sum_{p,q\in \Omega }\exp\left({\bar{V}}_{i,j}^{l,p,q}\right)} \end{array}$$

(18) $$\begin{array}{cc} {\tilde{X}}_{i,j}^{l} & =X_{i,j}^{l}+\sum_{p,q\in \Omega }{\hat{W}}_{i,j}^{l,p,q}\cdot X_{i,j}^{l} \end{array}$$

**Figure 9:**
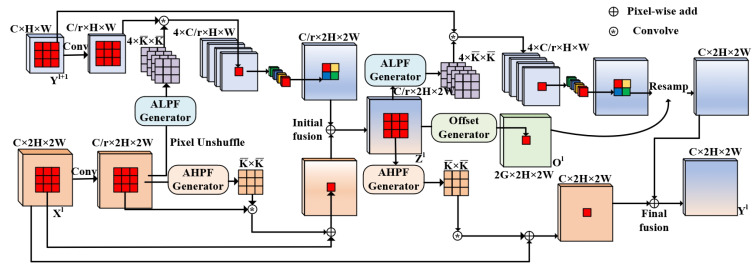
Overall structure of Adpfreqfusion.

In the above equations, Equations (10)–(13) provide the working principle of ALPF. First, ${\bar{V}}^{l}$ is obtained through a 3×3 convolution, then the feature values in the local area are normalized through the softmax operation to generate the weight ${\bar{W}}_{i,j}^{l,p,q}$. In this way, a low-pass filtering convolution kernel is obtained. After processing $Y_{i+p,j+q}^{l+1}$ with the convolution kernel, the final upsampled image ${\tilde{Y}}^{l+1}$ is obtained through PixelShuffle. Equation (14) is the working principle of the offset generator; by calculating the local cosine similarity $S_{i,j}^{l,p,q}$ to solve the problem of adjacent features having low intra-class similarity but high surrounding similarity, $S_{i,j}^{l,p,q}\in R^{8\times H\times W}$ stores the cosine similarity of each pixel and its eight neighboring pixels. This guidies the offset generator to sample features with high intra-class similarity, helping to reduce ambiguity in the inconsistent boundary regions. Equations (15)–(17) provide the working principle of AHPF. First, the feature map X(h,w) is transformed into the frequency domain through the Discrete Fourier Transform (DFT), where $X_{F}\left(u,v\right)\in R^{C\times H\times W}$ is the complex array output by the DFT and *h*, *w* are the coordinates of the feature map *X*. Then, in order to solve the situation of high-frequency components exceeding the Nyquist frequency being aliased or even lost during downsampling, $Z^{l}$ is used as the input to enhance the prediction of spatial-variable high-pass filters for the detailed boundary information lost during downsampling. Then, the low-pass kernel generated by softmax is subtracted from the unit kernel $E^{p,q}$ to obtain the high-pass kernel ${\hat{W}}_{i,j}^{l,p,q}$ and the result is finally obtained through residual enhancement output, as shown in Equation (18). The overall structure of the Adpfreqfusion module is shown in Figure 9.

#### 2.2.6. Lightweight Detection Head

The original detection head of YOLOv12 has limitations in recognition under conditions of limited illumination sampling and construction vibrations. First, the original detection head usually makes predictions using downsampled feature maps. This makes it difficult to identify densely stacked components in complex backgrounds, resulting in missed detections and false detections. Second, the original detection head relies on convolutional feature extraction. Under limited illumination sampling conditions, the signal-to-noise ratio of the feature map decreases, causing the image to blur. Moreover, the original detection head lacks relevant enhancement modules for the frequency domain, leading to classification errors and detection box position drift. In addition, images collected under the effect of construction vibration may exhibit dynamic blurring in the radial and longitudinal directions. The original detection head relies on convolution to extract local features, which damages key features such as the edges and textures of components. Furthermore, vibration can not only blur target features but also wrongly amplify high-frequency noise in the background, resulting in false detections. Therefore, the single-scale prediction structure of the original detection head restricts its dynamic learning ability, affecting object detection and resulting in low accuracy in situations when feature extraction is difficult.

**Figure 10:**
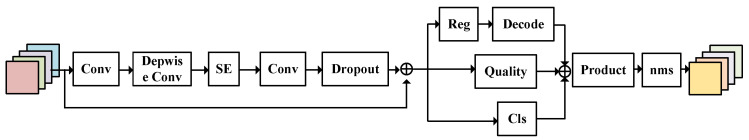
GFL-MBConv module structure.

MBConv was first proposed by Mark Sandler, Andrew Howard, et al. in 2018 [20]; however, its bounding box regression still uses the traditional Distribution-Focal Loss 1 (DFL1). Without a separate quality branch, it cannot model the confidence of the predicted distribution. To improve the detection performance of the model in such situations, this paper combines the MBConv (mobile inverted bottleneck convolution) module and proposes GFL-MBConv, as shown in Figure 10. The core idea of this innovative module is distribution modeling and quality perception. In addition, using the generalized focal loss for bounding box regression greatly accelerates the convergence speed.

Figure 10 shows how the GFL-MBConv module achieves efficient feature extraction and object detection through multistage processing. Its processing flow is as follows. First, a 1 × 1 point-wise convolution is adopted to expand the channels of the input features, increasing the feature dimensions to provide a richer information base. Subsequently, a depth-separable convolution structure is introduced, which consists of depth-wise convolution (DWConv) and point-wise convolution (PWConv). It first performs spatial feature extraction independently for each channel, then integrates cross-channel information through point-wise convolution. This design maintains a strong spatial feature extraction ability while significantly reducing computational complexity [21]. To further enhance the feature representation ability, a Squeeze-and-Excitation network (SE module) is embedded in the module. This module first preserves the original feature information through a residual connection to avoid the gradient degradation problem. In the squeezing stage, global average pooling is used to compress the spatial dimensions to 1 × 1 × C. In the excitation stage, a “bottleneck” structure (first reducing the dimension to 1 × 1 × C/r and then restoring to the original dimension) containing fully-connected, ReLU, and sigmoid layers is used to generate channel attention weights. Finally, adaptive feature enhancement in the channel dimension is achieved through feature recalibration [22]. The feature processing flow also includes using point-wise convolution to reduce the dimension in order to match the number of input channels, resulting in a reduced the number of parameters, as well as adding a dropout layer prior to the fully-connected layer in order to prevent overfitting by randomly deactivating neurons. The detection head part adopts a multi-branch design:

1. Distribution Regression Branch: A 1×1 convolution with 4×reg_max channels outputs a discrete probability distribution ([B,4,reg_max,H,W]), which is decoded into continuous coordinates through softmax and the distribution focal loss.
2. Distribution Quality Branch: Outputs a single-channel feature ([B,1,H,W]) and uses a sigmoid activation to evaluate the reliability of the prediction.
3. Classification Branch: Outputs the number of class channels ([B,num_classes,H,W]) and selects sigmoid or softmax activation according to the task requirements.

### 2.3. Dataset

During the trial operation of the scanning portal system, the AGV transported the shelf to the designated position and then stopped, at which point four cameras captured and stored images at a resolution of 600 × 600 pixels with a frame rate of 30 frames per second (fps). Subsequently, the videos were split and filtered frame-by-frame to ensure image clarity, ultimately generating the required dataset.

**Figure 11:**
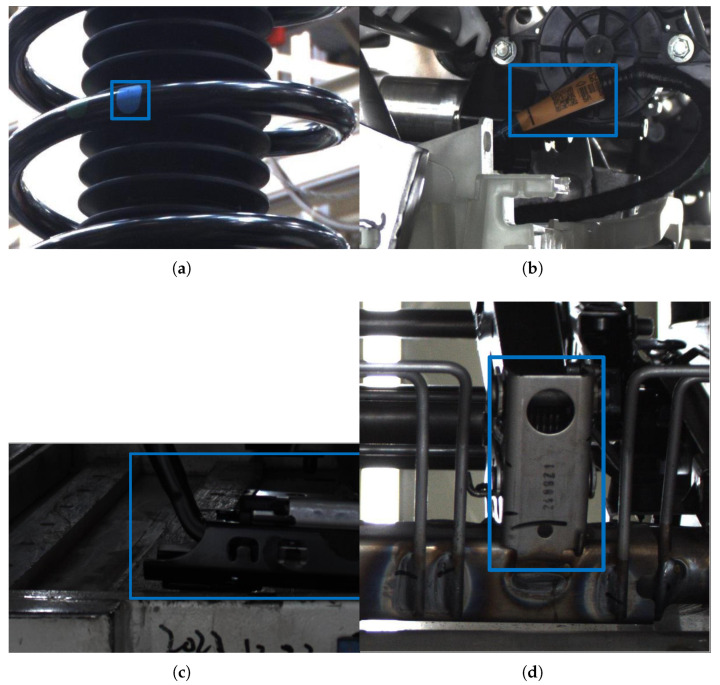
Examples of the dataset: (**a**) front suspension spring, (**b**) wiring harness port, (**c**) tie rod, (**d**) RZ.

The dataset includes four inspection targets, namely, label characters, color, and shape features, comprising a total of 10,915 images. Additionally, the dataset includes specific quantities for other components: the number of front suspension springs is 2145; the number of wiring harness ports is 2782; the number of tie rods is 3512; and the number of RZ components is 2476. Annotation was performed using X-AnyLabeling. To improve model generalization, random HSV perturbations were applied to the images: hue was adjusted by ±1.5%, saturation by ±70%, and value by ±40%. For geometric augmentation, random translations of up to ±10% in both horizontal and vertical directions, scaling by ±50%, and horizontal flipping with a 50% probability were implemented. These augmentations expanded the dataset to 32,745 images. The dataset was partitioned into training, validation, and test sets using a standard 80–10–10 split. A sample subset of the dataset is shown in Figure 11.

## 3. Results

### 3.1. Model Training

The experimental environment was configured with Windows 11 as the operating system, a 12th Gen Intel® Core™ i9-12900K processor (Intel Corporation, Santa Clara, CA, USA). processor running at 2.60 GHz, 128 GB of RAM, and a total disk capacity of 40 TB. The graphics processing unit consisted of four NVIDIA RTX 3090 GPUs with a combined 96 GB of VRAM.

For training the neural network model, PyTorch 1.12.3 was employed as the primary development framework. Each training iteration utilized a batch size of 32 samples, and the training process was conducted over 100 epochs. To ensure training stability and efficiency, the initial learning rate was set to 0.01 and the momentum was set to 0.8%. All other hyperparameters remained consistent throughout the experiments.

During the training process, the Stochastic Gradient Descent (SGD) optimizer was selected to optimize the network weights, aiming to achieve superior training results.

### 3.2. Evaluation Metrics

The evaluation metrics adopted in this study include the number of model parameters (Parameters), floating-point operations (FLOPs), precision (*P*), recall (*R*), average precision (*AP*), frames per second (FPS) and mean average precision (*mAP*). Here, True Positive (*TP*) refers to automotive component samples that are both detected and actually present; True Negative (*TN*) indicates samples where no automotive components are detected and none truly exist; False Positive (*FP*) represents instances where automotive components are detected but are non-existent; and False Negative (*FN*) denotes cases where automotive components exist but are undetected. *AP* denotes the average precision for a specific object category, while *mAP* represents the mean average precision across *k* object categories. The detailed computational methods for *A*, *AP*, *mAP*, *P*, and *R* are presented below.

(19) $$\begin{array}{c} A=\frac{TP+TN}{TP+TN+FP+FN} \end{array}$$

(20) $$\begin{array}{c} AP=\int_{0}^{1}P\left(R\right)\;dR \end{array}$$

(21) $$\begin{array}{c} mAP=\frac{1}{k}\sum_{i=1}^{k}AP_{i} \end{array}$$

(22) $$\begin{array}{c} P=\frac{TP}{TP+FP} \end{array}$$

(23) $$\begin{array}{c} R=\frac{TP}{TP+FN} \end{array}$$

### 3.3. Analysis of Experimental Results

#### 3.3.1. Ablation Study

To accurately evaluate the effectiveness of the improvement strategies, this paper adopts YOLOv12 as the baseline network and conducts ablation experiments on the collected automotive parts dataset mentioned above. The experimental results are shown in Table 1.

M1: Represents the experimental results of YOLOv12, where mAP@0.5 and mAP@0.5:0.95 are 0.9762 and 0.6899, respectively, with a parameter count of $2.52\times 10^{6}$, computational load of $6.0\times 10^{9}$, and FPS of 142. These serve as the baseline metrics for the experiments.
M2: Based on M1, the conventional convolution (Conv) is replaced with DYS2Dconv to enhance the network’s ability to recognize low-resolution targets. While the parameter count and computational load slightly decrease, the mAP@0.5, mAP@0.5:0.95, and FPS improve by 0.0088, 0.0601, and 16, respectively, demonstrating enhanced detection accuracy and efficiency.
M3: Building upon M2, the A2C2f_SA module is introduced to improve the network’s capability to locate features in complex backgrounds. With a reduction of $0.72\times 10^{6}$ in parameters and $1.2\times 10^{9}$ in computational load, mAP@0.5 and mAP@0.5:0.95 only decrease by 0.01 and 0.029, respectively, while the FPS increases further to 165.
M4: On the basis of M3, GFL-MBConv is employed as the new detection head to reduce the network’s parameters and computational load while maintaining detection accuracy as much as possible. With a slight reduction in parameters and computational load, mAP@0.5:0.95 decreases by only 0.008, while mAP@0.5 even increases by 0.005; in addition, the FPS reaches 183.
M5: Further improving upon M4, the feature fusion module in the neck is replaced with Adpfreqfusion to mitigate the loss of high-frequency information during model sampling. Compared to M1, the parameter count and computational load decrease to $1.02\times 10^{6}$ and $2.2\times 10^{9}$, respectively, while mAP@0.5 remains almost unchanged, mAP@0.5:0.95 improves by 0.0211, and the FPS increases to 195.

These experimental results demonstrate that the improved network architecture significantly reduces the model’s computational complexity while moderately enhancing its detection accuracy.

#### 3.3.2. Comparative Experiments on Different Attention Mechanisms

To validate the effectiveness of the proposed A2C2f-SA module, comparative experiments were conducted between A2C2f-SA and several commonly used attention mechanisms. It is worth noting that comparative experiments were also conducted on the value of the group number G. The experiments verified that its impact on performance is limited (fluctuations of R, MAP50, and MAP50-95 all less than 0.5%). Therefore, G was set to 8 by default to optimize computational efficiency.

As shown in Table 2, Model M1 represents the baseline model without any attention mechanism, while Models M2 to M5 incorporate the EMA [23], MBAttention [24], and FSAS [25] attention mechanisms, respectively. The experimental results demonstrate that the model equipped with A2C2f-SA achieves superior detection performance. Compared to the baseline model, A2C2f-SA improves mAP@0.5 and mAP@0.5:0.95 by 0.0078 and 0.0631, respectively, validating the effectiveness of the proposed attention mechanism.

#### 3.3.3. Comparative Experiments on Different Feature Fusion Modules

To validate the effectiveness of the proposed Adpfreqfusion feature fusion module, comparative experiments were conducted between Adpfreqfusion and several commonly used feature fusion modules.

As shown in Table 3, Model M1 represents the baseline configuration using YOLOv12’s native A2C2f and concatenation modules without enhancements, Model M2 incorporates the FPN [26] fusion module, Model M3 employs the BiFPN [27] module, and Model M4 demonstrates the proposed Adpfreqfusion implementation. Experimental results indicate that Adpfreqfusion achieves superior detection performance, improving mAP@0.5 and mAP@0.5:0.95 by 0.0006 and 0.0786, respectively, compared to the baseline, thereby verifying its advantages.

#### 3.3.4. Comparison of Indicators for Various Parts

The detection results for various components are presented in Table 4. The improved model demonstrates a significant reduction in model weight without compromising detection accuracy. Notably, it achieves a 0.013 increase in AP values for both the tie rod and RZ features, confirming that the model enhances detection precision while maintaining its lightweight characteristics.

#### 3.3.5. Comparison of Different Algorithms’ Detection Performance

To validate the effectiveness of the proposed algorithm, we conducted comparative experiments with current mainstream object detection algorithms, including SSD [28], Faster R-CNN [29], YOLOv8s, YOLOv10s, and YOLOv10n [30], and YOLOv11n and YOLOv11s. All experiments were performed under identical hardware and software conditions, with 10% of the dataset reserved for testing. The experimental results are presented in Table 5.

As shown in Table 5, the results demonstrate that our enhanced YOLO model achieves higher precision than all compared models except YOLOv11s, while maintaining only 0.135× the parameters of YOLOv11s and 0.34× the computational cost of YOLOv10n. Our enhanced YOLO model also demonstrates a significant advantage in FPS, achieving 195, markedly higher than competing models such as YOLOv11n and YOLOv10n, while also maintaining competitive mAP@0.5 and mAP@0.5:0.95. Compared to the other models in this experiment, our enhanced YOLO model is much more suitable for real-world deployment and automotive component detection.

#### 3.3.6. Visualization of Detection Results

**Figure 12:**
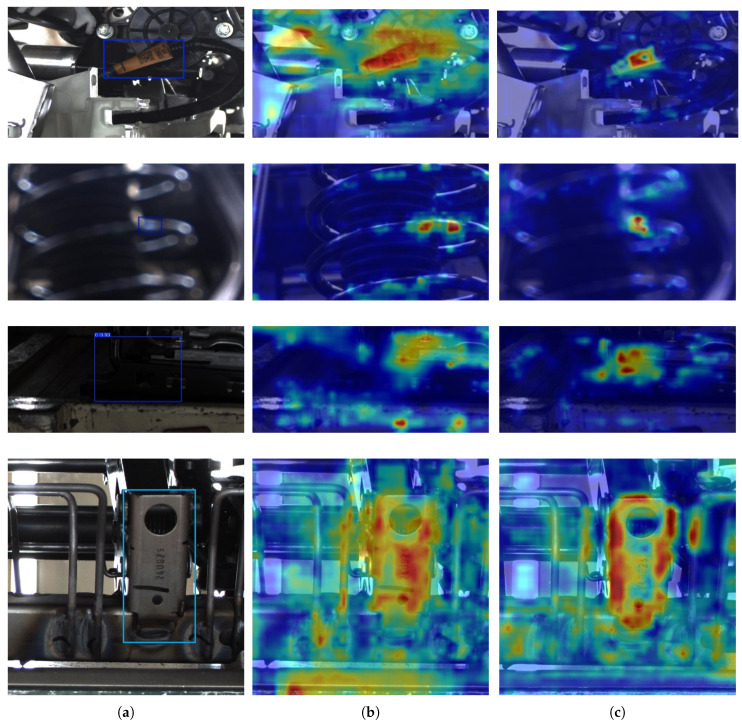
Comparative visualization of corresponding heatmaps. (**a**) The origin images. (**b**) Pre-A2C2f-SA integration. (**c**) Post-A2C2f-SA integration.

**Figure 13:**
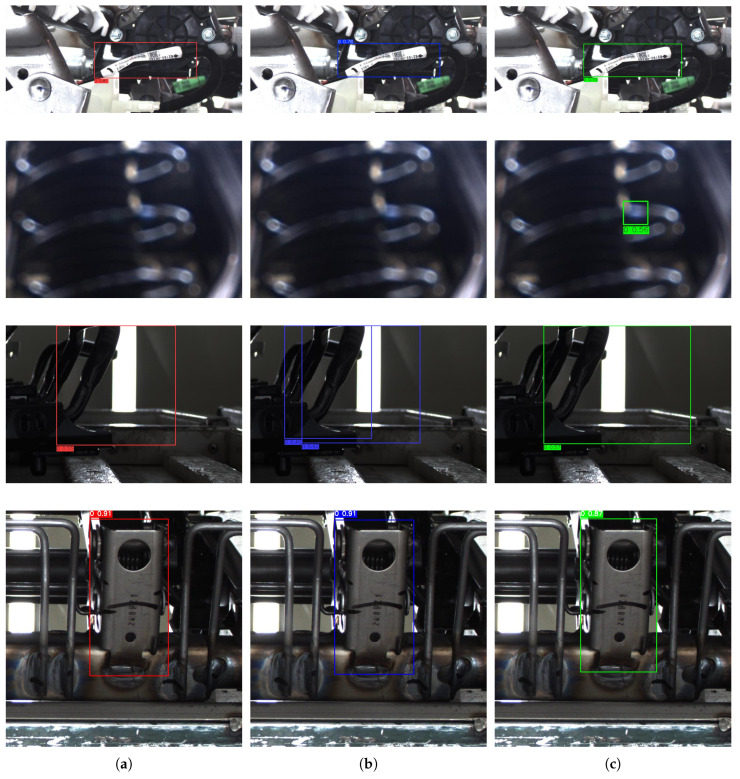
Visualizationof detection results. (**a**) YOLOv8n detection results. (**b**) YOLOv12n detection results. (**c**) Enhanced YOLOv12n detection results.

- A2C2f-SA Heatmap Visualization: To intuitively demonstrate the impact of A2C2f-SA on detection accuracy, we employ heatmap visualization to compare feature extraction sensitivity before and after incorporating the A2C2f-SA module.
  As shown in Figure 12, comparative visualization of the corresponding heatmaps pre- and post-A2C2f-SA integration reveals that the model’s attention becomes more concentrated on target objects after implementing A2C2f-SA, demonstrating enhanced capability in learning discriminative features from raw images.
- Detection Results Visualization: To visually demonstrate the detection performance of the enhanced YOLO, we compared its results with YOLOv8n and YOLOv12n on automotive component dataset images.
  As shown in Figure 13, when processing low-quality images with a confidence threshold of 0.5, YOLOv8n and YOLOv12n fail to detect the target objects, while our enhanced YOLO model successfully identifies them, demonstrating superior robustness. Although our enhanced YOLO shows slightly lower precision in harness port detection due to its reduced parameter count and computational complexity, it achieves higher confidence scores than YOLOv8n in tie rod detection and avoids the multiple bounding box issue observed in YOLOv12n.

#### 3.3.7. Actual Field Operation Test

To test the actual performance of the proposed scanning portal system and enhanced YOLOv12 model, we conducted a comprehensive on-site test of the system under conditions which closely resembled actual industrial operations. The automotive components to be detected consisted of front suspension springs, wiring harness ports, tie rods, and RZ. The test lasted for 4 days, with an average of 6 h of testing per day. A total of 294 AGV transport racks were detected, and the total number of parts was 5880 pieces. The final results are shown in Table 6.

As can be seen from Table 6, the total accuracy of the proposed system is 97.3% and the average detection time is 7.59 s. During the actual detection process, the system runs smoothly and can meet industrial requirements.

## 4. Discussion

To enhance the accuracy and operational efficiency in the outbound logistics of automotive component, an online detection system based on YOLOv12n and a scanning portal system is designed in this paper. The enhanced YOLOv12 model employed in this study achieves lightweight performance with minimal sacrifice of detection accuracy while improving precision. This enhancement boosts detection efficiency and reduces labor costs. The focus of this section is to discuss the research findings.

This paper proposes an improved YOLOv12 object detection model that enhances performance through multi-module collaborative optimization. In the backbone network, a lightweight A2C2f-SA attention module and Dynamic Space-to-Depth (Dynamic S2D) convolution are introduced to strengthen feature representation while minimizing information loss. A GFL-MBConv lightweight detection head is designed to improve inference speed, and an adaptive frequency-aware feature fusion module (Adpfreqfusion) is integrated into the neck network to restore high-frequency details through frequency domain feature fusion, significantly improving detection accuracy in complex scenarios. This method achieves a balance between accuracy and speed while maintaining high efficiency.

Ablation experiments demonstrate the effectiveness of the proposed modules: replacing standard convolution and pooling layers with DYS2Dconv improves mAP@0.5, mAP@0.5:0.95, and FPS by 0.88%, 6.01%, and 16, respectively; introducing the A2C2f-SA module reduces parameters by 36% with only a 1–2.9% drop in accuracy; adopting the GFL-MBConv detection head maintains detection precision while further reducing model complexity; finally, the Adpfreqfusion module reduces parameters by 59.5% while improving mAP@0.5:0.95 and FPS by 2.11% and 53 over the baseline, respectively. These modules work synergistically to significantly reduce model complexity while enhancing detection accuracy.

Comparative experiments on different attention mechanisms show that the A2C2f-SA module outperforms common attention mechanisms such as EMA, MBAttention, and FSAS, improving mAP@0.5 and mAP@0.5:0.95 by 0.78% and 6.31% over the baseline model, respectively. This validates the effectiveness of A2C2f-SA in optimizing feature attention mechanisms and significantly enhancing object detection accuracy. What is more, the negligible impact of the group size G on performance further demonstrates the module’s robustness to normalization granularity, making it practical for deployment without the need to fine-tune hyperparameters.

Experiments with different feature fusion modules demonstrate that the proposed Adpfreqfusion module surpasses traditional methods such as FPN and BiFPN, increasing mAP@0.5 and mAP@0.5:0.95 by 0.06% and 7.86% over the baseline, respectively. The module effectively improves detection accuracy through adaptive frequency domain feature fusion, confirming its superiority.

A comparative study between the proposed algorithm and the original YOLOv12n as well as other mainstream algorithms revealed that our enhanced YOLO model achieves superior detection performance while maintaining lightweight characteristics; its parameter count is only 13.5% of YOLOv11s and its computational load is reduced to 34% of YOLOv11n, yet it outperforms mainstream algorithms such as SSD, Faster R-CNN, and the YOLOv8s/v10 series in terms of accuracy. The proposed model demonstrates significant advantages in automotive part detection, meeting the lightweight requirements for industrial deployment while ensuring high detection accuracy.

Visualization of the experimental results confirms that incorporation of the A2C2f-SA module enhances the model’s focus on target regions, improving its feature extraction capability. Comparative tests show that our enhanced YOLO model successfully detects blurred targets missed by YOLOv8n and YOLOv12n at a confidence threshold of 0.5, demonstrating stronger robustness. Although slightly less precise in detecting wire harness ports, our enhanced YOLO exhibits unique advantages in automotive part detection, validating its practical applicability by avoiding multiple bounding box issues and achieving higher confidence in detecting components such as pull rods.

On-site tests indicate that the proposed system achieves a total accuracy rate of 97.3%, with an average detection time of the transport rack of 7.59 s; in contrast, manual detection exhibits an accuracy rate below 95% and the average detection time per transport rack exceeds 40 s. Compared to manual methods, the proposed system demonstrates an improvement in accuracy by more than 2.3% and an increase in detection efficiency by approximately 81.03%, highlighting its superior performance.

In conclusion, the proposed algorithm fully meets the requirements for object detection in automotive part scenarios, achieving lightweight performance with minimal compromise in detection accuracy.

## 5. Conclusions

Application of the proposed machine vision-based intelligent recognition and detection system can significantly enhance accuracy and operational efficiency in the outbound logistics of automotive components. By leveraging multi-angle industrial cameras for image acquisition along with barcode recognition and AI-powered real-time detection technologies, the proposed system ensures that parts on shelves are perfectly aligned with production orders while enabling seamless data interaction with the Warehouse Management System (WMS). In case of anomalies, the system promptly issues alerts and generates records, with a backup scanning solution ensuring the continuity of production processes. This solution not only eliminates misplacement and omission errors but also establishes a comprehensive data traceability system, providing highly reliable quality assurance for automotive parts logistics. Its successful implementation demonstrates the significant value of intelligent technologies in industrial logistics, offering a reusable technical paradigm for similar scenarios and laying a solid foundation for further optimization of warehouse and logistics automation.

In terms of detection methodology, the proposed system transitions from periodic sampling to round-the-clock real-time monitoring. Field results demonstrated an accuracy rate of 97.3%, effectively preventing misplacement and omission issues. In addition, the average inspection time for each AGV is 7.59 s, which can significantly improve the efficiency of automotive parts inspection.

However, the current system still exhibits certain limitations that constrain its operational flexibility. The primary constraint originates from a combination of dataset scope and model architecture: although the system encompasses most standard automotive components, its static architecture is incapable of recognizing newly introduced parts that were not present in the original training data. This dual limitation results in two critical operational challenges: (1) during outbound inspection of novel components, the system requires manual intervention for unrecognized parts; and (2) integration of new part types requires complete model retraining, leading to operational delays in dynamic industrial environments. Collectively, these challenges motivate and underscore the necessity for our planned implementation of online learning capabilities and architectural enhancements aimed at enabling continuous adaptation.

Looking ahead, we will continue to upgrade the scanning portal system and optimize its algorithms. For instance, based on the latest algorithmic modules introduced this year, more suitable feature fusion modules and attention mechanisms will be explored to further improve detection accuracy and real-time performance. Additionally, integrating multimodal data fusion technologies such as infrared, LiDAR, and 3D point clouds with the proposed scanning portal system could help to enhance system robustness. Furthermore, online learning will be introduced to improve scalability, with augmented images collected from the field uploaded to a data stream to enable the model to continuously update and adapt to new data in real time. These enhancements will further the goal of achieving more efficient deployment and application in the field of automotive parts recognition.

## Figures and Tables

**Table 1:** Ablation study results of different model configurations.

Model	Base	Spdconv	A2C2f- SA	GFL- MB Conv	Adpfreq Fusion	mAP@0.5	mAP@ 0.5:0.95	Params /$\times 10^{6}$	FLOPs /$\times 10^{9}$	FPS
M1	✓					0.9762	0.6899	2.52	6.0	142
M2	✓	✓				0.9850	0.7500	2.17	5.4	158
M3	✓	✓	✓			0.9750	0.7210	1.45	4.2	165
M4	✓	✓	✓	✓		0.9800	0.7130	1.13	2.9	183
M5	✓	✓	✓	✓	✓	0.9739	0.7110	1.02	2.2	195

**Table 2:** Performance comparison of attention mechanisms.

Model	Attention Mechanism	Key Improvement	mAP@0.5	mAP@ 0.5:0.95
M1	None	Baseline without any attention mechanism	0.9762	0.6899
M2	EMA	Efficient multi-scale attention with lightweight cross spatial-channel interactions	0.9760	0.7440
M3	MBAttention	Multi-branch parallel pathways for enhanced feature diversity	0.9780	0.7320
M4	FSAS	Joint frequency–spatial modeling with adaptive sampling	0.9810	0.7470
M5	A2C2f-SA	Channel and spatial attention fusion with shuffle mechanism	0.9840	0.7530

**Table 3:** Performance comparison of feature fusion modules.

Model	Feature Fusion Module	Key Improvement	mAP@0.5	mAP@ 0.5:0.95
M1	None	Baseline without any feature fusion module	0.9762	0.6899
M2	FPN	Enhanced multi-scale feature interaction through bidirectional cross-scale feature fusion	0.9759	0.7032
M3	BiFPN	Enhanced multi-scale feature fusion via weighted bidirectional cross-scale connections and cross-node feature reuse	0.9686	0.7354
M4	Adpfreqfusion	Control over the ratio of high/low-frequency components through learnable gating	0.9768	0.7685

**Table 4:** AP values of different models for various component types.

Component Type	AP			
	Front Suspension Spring	Wiring Harness Port	Tie Rod	RZ
YOLOv12n	0.994	0.96	0.973	0.977
Enhanced YOLO	0.987	0.958	0.986	0.990

**Table 5:** Performance comparison of different models.

Model	Param/$10^{6}$	FLOPs/$10^{9}$	P	R	mAP@0.5	mAP@0.5:0.95	FPS
SSD	12.3	38.8	0.948	0.945	0.877	0.645	48
Faster R-CNN	32.1	39.5	0.953	0.964	0.9542	0.682	32
YOLOv8s	11.8	12.6	0.974	0.985	0.984	0.741	122
YOLOv10s	7.2	21.6	0.979	0.983	0.985	0.746	138
YOLOv10n	2.708	8.2	0.971	0.977	0.984	0.733	165
YOLOv11n	2.6	6.5	0.973	0.978	0.983	0.732	170
YOLOv11s	9.4	21.5	0.981	0.987	0.986	0.742	147
Enhanced YOLO	1.272	2.2	0.98	0.97	0.9739	0.711	195

**Table 6:** Detection results.

Component Type	Quantity of Parts/Piece	Accuracy/%	Average Detection Time per Rack/s
Front Suspension Spring	1240	97.7	6.2
Wiring Harness Port	1240	98.9	5.8
Tie Rod	1700	97.6	7.9
RZ	1700	95.5	8.6
Total	5880	**97.3**	**7.59**

## Data Availability

The data are available upon request to the correspondence e-mail.
